# Contact lens-related versus non-contact lens–related *Pseudomonas aeruginosa* keratitis: The Midlands Infectious Keratitis Study

**DOI:** 10.3389/fmed.2026.1835834

**Published:** 2026-07-10

**Authors:** Burak Ozturk, Saba Anwar, Khawaja Muhammad Ammar Ali Javed, Yu Jeat Chong, Gibran Butt, Liying Low, Dalia G. Said, Harminder S. Dua, Ankur Barua, Anil Aralikatti, Saaeha Rauz, Darren S. J. Ting

**Affiliations:** 1 Birmingham and Midland Eye Centre, Sandwell and West Birmingham NHS Trust, Birmingham, United Kingdom; 2 Academic Unit of Ophthalmology, Department of Inflammation and Ageing, School of Infection, Inflammation and Immunology, College of Medicine and Health, University of Birmingham, Birmingham, United Kingdom; 3 Department of Ophthalmology, Queen’s Medical Centre, Nottingham, United Kingdom; 4 Academic Ophthalmology, School of Medicine, University of Nottingham, Nottingham, United Kingdom; 5 National Eye Centre, Singapore Eye Research Institute, Singapore, Singapore; 6 Ophthalmology and Visual Sciences Academic Clinical Program, Duke-NUS Medical School, Singapore, Singapore

**Keywords:** contact lens, corneal infection, corneal ulcer, microbial keratitis, risk factors

## Abstract

**Background:**

*Pseudomonas aeruginosa* (PA) is a major cause of infectious keratitis worldwide. Contact lens (CL) wear is a key risk factor, though other predisposing factors exist. This study compared clinical features, risk factors, and outcomes of CL-related versus non-CL-related PA keratitis in the United Kingdom (UK).

**Methods:**

A retrospective cohort study of culture-confirmed PA keratitis cases that presented to the Birmingham and Midland Eye Centre (Birmingham, UK) and Queen’s Medical Centre (Nottingham, UK) between August 2016 and January 2020. Demographics, clinical characteristics, risk factors, and outcomes were analysed and compared between CL-related and non-CL-related groups.

**Results:**

Sixty-six patients (66 eyes) were included; mean age was 54.0 ± 20.9 years and 59.1% were female. CL wear accounted for 35 (53.1%) cases. At presentation, mean CDVA was 1.26 ± 0.96 logMAR (~6/120 Snellen). Hospitalisation occurred in 71.2% cases, with 19.7% cases requiring surgery. Most isolated PA (64, 97.0%) were antibiotic-susceptible. Compared to non-CL-related keratitis, CL-related keratitis was more commonly associated with younger age, better initial vision, smaller and non-central ulcers, and absence of hypopyon (all *p* < 0.05). CL-related cases had a significantly lower risk of poor visual outcome (>0.6 logMAR; adjusted odd ratio = 0.13, 95% CI 0.01–0.84, *p* = 0.046; multivariable logistic regression) and faster rate of corneal healing (median: 17 days vs. 32 days; *p* = 0.029; unadjusted Kaplan–Meier analysis).

**Conclusion:**

PA keratitis remains a significant cause of ocular morbidity in the United Kingdom. CL-related disease shows distinct clinical features and better outcomes likely due to lower presenting disease severity and timely management of the disease.

## Introduction

Infectious keratitis (IK) is the leading cause of corneal blindness worldwide, particularly in low- and middle-income countries where timely access to ophthalmic care is often limited (1–3). In high-income nations, including the United Kingdom (UK), bacterial keratitis accounts for around 90% of all IK cases, with *Pseudomonas aeruginosa* (PA) consistently identified as one of the most common causative organisms (4–7).

PA is a Gram-negative, aerobic, rod-shaped bacterium found in soil, water and hospital environments such as handwashing sinks, ventilator circuits, water systems, and medical devices. Its pathogenicity is driven by multiple virulence mechanisms, including the secretion of proteases and exotoxins that damage epithelial tissues, production of pigments and enzymes that disrupt host immunity, and biofilm formation that confers resistance to antibiotics (8). PA is recognised as a key ESKAPEE (*Enterococcus faecium, Staphylococcus aureus, Klebsiella pneumoniae, Acinetobacter baumannii, Pseudomonas aeruginosa, Enterobacter species, Escherichia coli*) pathogens and was amongst the leading causes of antimicrobial resistance (AMR)-related deaths globally in 2019 (9, 10).

In ocular infections, PA is known as one of the most destructive bacterial pathogens, capable of causing fulminant corneal ulcers and rapid visual loss if treatment is delayed (8). Despite prompt and intensive therapy, complications such as corneal melt, perforation, and endophthalmitis may occur, leading to significant visual morbidity and reduced quality of life. Contact lens (CL) wear is the predominant risk factor for PA keratitis in developed countries (11, 12), although other factors such as ocular surface disease, trauma, post-corneal surgery, and systemic immunosuppression can also contribute (11, 12).

To date, only one study from the United States has directly compared the characteristics and outcomes of CL-related and non-CL-related PA keratitis (13), and no such study been conducted elsewhere, including the United Kingdom. Recent United Kingdom studies highlighted regional variations in incidence and microbial profiles, with PA being consistently (albeit not universally) identified as the leading pathogen (4, 11, 14–16).

To address the knowledge gap, we conducted a comparative analysis of culture-positive CL-related and non-CL-related PA keratitis across two United Kingdom tertiary ophthalmic referral centres in the Midlands region, which serves a highly diverse population (17, 18). We hypothesised that PA keratitis in the United Kingdom manifests as two clinically distinct phenotypes, with differing presentations and outcomes.

## Materials and methods

### Study design and ethical approval

This was a retrospective study of culture-positive PA keratitis cases that presented to the Queen’s Medical Centre (QMC), Nottingham, United Kingdom, and the Birmingham and Midland Eye Centre (BMEC), Birmingham, United Kingdom, between August 2016 and January 2020. Study approval was obtained from the Clinical Governance teams at Nottingham University Hospitals NHS Trust (Ref No: 19–265C) and Sandwell and West Birmingham NHS Trust (Ref No: 2444) as clinical effectiveness audits. The study adhered to the principles of the Declaration of Helsinki.

### Case identification and eligibility

All potential cases of IK that had undergone corneal sampling were identified via the local microbiology databases of Nottingham and Birmingham. Electronic medical records of the included cases were reviewed, and only those with culture-positive PA keratitis with complete initial and follow-up data were included. In bilateral cases, only one eye per patient was randomly included to maintain statistical independence. For recurrent cases, only the first episode of infection was considered.

### Data collection

All relevant data, including demographic factors, ocular and systemic risk factors, duration of symptoms prior to presentation, clinical features, microbiological results, clinical outcomes, medical and/or acute surgical management, and complications (if any), were extracted and recorded using a standardised excel proforma, similar to the previous studies (11, 14). Risk factors were categorised into CL wear, trauma, ocular surface disease (e.g., dry eye, blepharitis, limbal stem cell deficiency, cicatricial conjunctival disease, exposure keratopathy, neurotrophic keratopathy, and band keratopathy), previous corneal surgery (e.g., keratoplasty), systemic immunosuppression (e.g., diabetes and those on systemic immunosuppressive drugs), and recent use of topical corticosteroids. The size of epithelial defect and infiltrate was classified as small (≤3 mm), moderate (3.1–6 mm), or large (>6 mm) based on the maximum linear dimension. The location of ulcer was categorised as central (any part of the ulcer affecting the visual axis), paracentral (in between central and peripheral), and peripheral (the entire ulcer within 3 mm from the limbus). The main outcome measures were corrected distance visual acuity (CDVA) at the final follow-up visit and time to complete corneal healing.

### Clinical management

According to the local IK guidelines, all patients who presented with corneal infiltrates >1 mm in diameter, central ulcer, or atypical manifestations of corneal ulcers underwent corneal sampling for microbiological investigations. Corneal sampling was performed using either hypodermic needles or corneal swabs, and the samples were sent for microscopy (with Gram staining), culture and/or polymerase chain reaction (PCR), and susceptibility testing (11, 19). Contact lens were sent for microbiological investigation in some cases, but the management of IK was primarily guided by the corneal scrape results. Chocolate, blood and Sabouraud agar plates were used for culturing bacterial and fungal pathogens (with at least 1 week of incubation period). Only culture-positive PA cases were included. Depending on the clinical severity and clinician preference, the first-line topical antibiotic therapy included levofloxacin 0.5% monotherapy or fortified cefuroxime 5% combined with either gentamicin 1.5% or amikacin 2.5% combination therapy. Subsequent modification of the topical and/or systemic antimicrobial agents might be indicated depending on microbiological results and treatment response. Hospital admission was indicated for severe or refractory corneal ulcers or patients where adherence to intensive treatment was unlikely. Acute surgical interventions, including corneal gluing, amniotic membrane transplant, tectonic/therapeutic keratoplasty, and/or anophthalmic surgery, were performed in some cases where clinically indicated (e.g., corneal perforation or uncontrolled infection).

### Statistical analysis

All analyses were performed using IBM SPSS Statistics version 29.0 (IBM Corp., Armonk, NY, United States). For descriptive and analytic purposes, cases were divided into CL-related and non-CL-related PA keratitis. Continuous variables were summarised as mean ± standard deviation (SD) for normally distributed data and as median (interquartile range [IQR]) for non-normal data. Categorical variables were expressed as frequencies (%). Normality was assessed using the Shapiro–Wilk test. Chi-square/Fisher’s exact was used to analyse categorical data whereas independent-samples *t*-test/Mann–Whitney *U* test was used for group comparisons for normal or non-normal continuous variables, respectively. Statistical significance was defined as two-sided *p* < 0.05.

The primary outcome was final CDVA measured at the final follow-up visit, defined as good (≤0.6 logMAR) or poor (>0.6 logMAR), and secondary outcomes included the time to complete corneal healing, hospitalisation, surgical intervention. Time to complete corneal healing was defined as time taken from initial presentation to complete corneal epithelialisation and resolution of infection. Univariable logistic regression was first used to assess potential predictors for poor visual outcome (final CDVA >0.6 logMAR or worse than 6/24 Snellen vision) and delayed corneal healing (i.e., >30 days to achieve complete corneal healing from the initial presentation, occurrence of uncontrolled infection or corneal perforation requiring corneal gluing, tectonic or therapeutic keratoplasty, and/or evisceration/enucleation). These thresholds were used as exploratory binary cut-offs to support prognostic modelling, similar to previous studies (11, 14). Kaplan–Meier analysis retained time to complete corneal healing as the time-to-event endpoint.

Potential significant variables (with *p* < 0.10) or with clinical relevance were then entered into multivariable logistic regression models. Adjusted odds ratios (aORs) with 95% confidence intervals (CIs) were reported. Kaplan–Meier survival analysis was used to compare the time to complete corneal healing between CL-related and non-CL-related cases, stratified by the presenting infiltrate size (≤3 mm vs. >3 mm. Differences between the curves were tested using the log-rank test. Median healing times were calculated from survival estimates.

## Results

### Overall description

This study included 66 patients (*n* = 66 eyes) with culture-positive PA keratitis (Table 1). The mean age was 54.0 ± 20.9 years, with 81.8% White ethnicity and a female predominance (59.1%). There were 35 (53.1%) and 31 (46.9%) cases of CL-related and non-CL-related PA keratitis, respectively. CL wear was the most common risk factor (35, 53.1%), followed by ocular surface disease (23, 34.8%), systemic immunosuppression (14, 21.2%), and previous corneal surgery (8, 12.1%). Of the 14 patients with systemic immunosuppression, 8 had diabetes mellitus and 6 were on systemic immunosuppressive drugs. Six (9.1%) patients had no identifiable risk factors.

**Table 1:** Summary of demographic, clinical characteristics and risk factors of patients with contact lens (CL) and non-CL-related *Pseudomonas aeruginosa* keratitis.

Parameter	All cases (*N* = 66) *N* (%)	CL (*N* = 35) *N* (%)	Non-CL (*N* = 31) *N* (%)	*p*-value^*^
Age (years)	54.0 ± 20.9	41.5 ± 17.1	70.5 ± 14.4	**<0.001**
Ethnicity				0.20
White	54 (81.8)	28 (80.0)	26 (83.8)	
Black	6 (9.1)	2 (5.7)	4 (12.9)	
Asian	6 (9.1)	5 (14.3)	1 (3.3)	
Gender, female	39 (59.1)	20 (57.1)	19 (61.3)	0.93
Affected eye, left eye	40 (60.6)	23 (65.7)	17 (54.8)	0.52
Risk factors^**^				
Ocular surface disease	23 (34.8)	4 (11.4)	19 (61.3)	**<0.001**
Systemic immunosuppression^***^	14 (21.2)	3 (8.5)	11 (36.6)	**0.014**
Previous corneal surgery	8 (12.1)	1 (2.8)	7 (22.5)	**0.021**
Trauma	2 (3.0)	1 (2.8)	1 (3.2)	1.00
Topical corticosteroid use	4 (6.0)	0 (0.0)	4 (12.9)	**0.009**
Symptom duration before presentation	2.6 ± 1.7	2.3 ± 1.8	2.9 ± 1.5	0.14
Presenting CDVA (logMAR)	1.26 ± 0.96	0.82 ± 0.87	1.74 ± 0.82	**<0.001**
<0.3	17 (25.9)	15 (42.9)	2 (6.5)	
0.31–0.6	9 (13.6)	7 (20.0)	2 (6.5)	
0.61–1.0	9 (13.6)	3 (8.6)	6 (19.3)	
>1.0	31 (46.9)	10 (28.5)	21 (67.7)	
Size of epithelial defect (mm)				**0.008**
≤3 (small)	28 (42.4)	21 (60.0)	7 (22.6)	
3.1–6 (moderate)	26 (39.4)	10 (28.6)	16 (51.6)	
>6 (large)	12 (18.2)	4 (11.4)	8 (25.8)	
Size of infiltrate (mm)				**0.002**
≤3 (small)	32 (48.5)	24 (68.6)	8 (25.8)	
3.1–6 (moderate)	23 (34.8)	8 (22.8)	15 (48.4)	
>6 (large)	11 (16.7)	3 (8.6)	8 (25.8)	
Location of ulcer				**0.001**
Central	27 (40.9)	8 (22.8)	19 (61.3)	
Paracentral	28 (42.4)	17 (48.6)	11 (35.5)	
Peripheral	11 (16.7)	10 (28.6)	1 (3.2)	
Hypopyon (Present)	33 (50.0)	13 (37.1)	20 (64.5)	**0.048**
Hospitalisation (Yes)	47 (71.2)	22 (62.9)	25 (80.6)	0.18

^*^Comparison was made between CL-related and non-CL-related cases. Significant *p*-values (<0.05) are in bold.

^**^Some patients had more than one risk factors.

^***^Systemic immunosuppression includes diabetes (total *n* = 8; 2 in CL group vs 6 in non-CL group) and those on systemic immunosuppression drugs (total *n* = 6; 1 in CL group vs. 5 in non-CL group).

All categorical values are presented as number and (percentage in bracket), and continuous values are presented as mean ± standard deviation (SD).

CDVA, Corrected distance visual acuity; CL, Contact lens.

At presentation, the mean CDVA was 1.26 ± 0.96 logMAR, and 31 (46.9%) patients had poor presenting vision (CDVA >1.0 logMAR). The majority of the cases had small epithelial defect size (28, 42.4%), small infiltrate size (32, 48.5%), paracentrally located ulcer (28, 42.4%), and presence of hypopyon (33, 50%) (Table 1). The mean symptom duration prior to the presentation was 2.6 ± 1.7 days.

### Antimicrobial susceptibility results

Antimicrobial susceptibility results were available for amikacin in 39 isolates, gentamicin in 55 isolates, and ciprofloxacin in 53 isolates. Susceptibility was high for all three agents: 39/39 (100.0%) isolates were susceptible to amikacin, 54/55 (98.2%) to gentamicin, and 52/53 (98.1%) to ciprofloxacin. Only one case of gentamicin-resistant PA (CL-related) and one case of ciprofloxacin-resistant PA (non-CL-related) were noted in this study (Table 2).

**Table 2:** Antimicrobial susceptibility results of *Pseudomonas aeruginosa* isolated from patients with contact lens (CL)-related and non-CL-related *Pseudomonas aeruginosa* keratitis.

Parameter	All cases	CL-related	Non-CL-related	*p*-value*
Amikacin	S: 39/39 (100.0%); R: 0/39 (0.0%)	S: 22/22 (100.0%); R: 0/22 (0.0%)	S: 17/17 (100.0%); R: 0/17 (0.0%)	1.000
Gentamicin	S: 54/55 (98.2%); R: 1/55 (1.8%)	S: 30/31 (96.8%); R: 1/31 (3.2%)	S: 24/24 (100.0%); R: 0/24 (0.0%)	1.000
Ciprofloxacin	S: 52/53 (98.1%); R: 1/53 (1.9%)	S: 31/31 (100.0%); R: 0/31 (0.0%)	S: 21/22 (95.5%); R: 1/22 (4.5%)	0.42

^*^Susceptibility *p*-values compare resistance proportions between CL-related and non-CL-related cases using Fisher’s exact test.

S, Susceptible; R, Resistance (also included intermediate).

Antimicrobial denominators differ because susceptibility results were not test for all 3 antibiotics for every isolate.

### Comparison between CL-related and non-CL-related PA keratitis

CL-related PA keratitis occurred in significantly younger patients than non-CL-related cases (41.5 ± 17.1 vs. 70.5 ± 14.4 years; *p* < 0.001) (Table 1). Gender and laterality did not differ between groups. The mean symptom duration before presentation was borderline shorter in CL-related cases (2.3 ± 1.8 days) than non-CL-related cases (2.9 ± 1.5 days), but the difference was not statistically significant (*p* = 0.13; Table 1).

CL-related cases presented with better CDVA (0.82 ± 0.87 logMAR vs. 1.74 ± 0.82 logMAR; *p* < 0.001), smaller epithelial defects (≤3 mm in 60.0% vs. 22.6%, *p* = 0.008), and smaller infiltrates (≤3 mm in 68.6% vs. 25.8%, *p* = 0.002), and were less likely to have central ulcers (22.8% vs. 61.3%, *p* = 0.001) and hypopyon (37.1% vs. 64.5%, *p* = 0.048; Table 1).

### Clinical management and outcomes

Hospitalisation was required in 47 (71.2%) cases, with a higher rate amongst non-CL-related cases than CL-related cases (80.6% vs. 62.9%), though not statistically significant (*p* = 0.18; Table 1). Most cases (58, 87.9%) were controlled medically, while 8 patients (12.1%) required at least one acute surgical intervention, including corneal gluing (6, 9.1%), amniotic membrane transplantation (1, 1.5%), and evisceration (1, 1.5%) (Table 3). Elective optical penetrating keratoplasty was performed at a later stage in 3 (4.5%) patients after a complete resolution of infection. Although acute surgical intervention was more frequent amongst non-CL-related ulcers (19.4% vs. 5.7%), this difference was not statistically significant (*p* = 0.13; Table 3).

**Table 3:** Clinical outcomes and acute surgical management of patients with *Pseudomonas aeruginosa* keratitis.

Parameter	All cases *N* = 66, *N* (%)	CL-related *N* = 35, *N* (%)	Non-CL-related, *N* = 31, *N* (%)	*p*-value^*^
Presenting CDVA, logMAR	1.26 ± 0.95	0.82 ± 0.87	1.74 ± 0.82	**<0.001**
Final CDVA, logMAR	0.76 ± 0.96	0.27 ± 0.40	1.32 ± 0.78	**<0.001**
Improvement in CDVA, logMAR	0.49 ± 1.00	0.55 ± 0.97	0.42 ± 1.05	**<0.001**
Good final CDVA (≤0.6 logMAR)	46 (69.7)	33 (94.3)	13 (41.9)	**<0.001**
Acute surgical intervention (yes)	8 (12.1)	2 (5.7)	6 (19.4)	0.13
Corneal gluing	6	1 (2.9)	5 (16.1)	
AMT	1	1 (2.9)	0	
Evisceration	1	0 (0.0)	1 (3.2)	
Delayed corneal healing^**^	29 (43.9)	11 (31.4)	18 (58.1)	**0.030**

^*^Comparison was made between CL-related and non-CL-related cases using unpaired or paired *T*-test for mean difference, and Fisher-exact test of Chi-square test for proportion difference. Significant *p*-values (<0.05) are in bold.

**Delayed corneal healing is defined as >30 days to achieve complete corneal healing from the initial presentation, occurrence of uncontrolled infection or corneal perforation requiring corneal gluing, tectonic or therapeutic keratoplasty, and/or evisceration/enucleation.

AMT, Amniotic membrane transplant; CDVA, Corrected-distance-visual-acuity; CL, Contact lens.

Categorical values are presented as percentage (in bracket) and continuous values are presented as mean ± standard deviation (SD).

Final CDVA improved significantly across the cohort from 1.26 ± 0.96 logMAR at presentation to 0.76 ± 0.82 logMAR at final follow-up, with a mean improvement of 0.49 ± 1.00 logMAR (95% CI 0.25–0.74; *p* < 0.001; paired t-test). Patients with CL-related keratitis achieved significantly better final CDVA than those with non-CL-related infections (0.27 ± 0.40 vs. 1.32 ± 0.78 logMAR; *p* < 0.001). Good visual outcome (≤0.6 logMAR) was achieved in 46 (69.7%) patients overall, with a significantly higher proportion in CL-related cases (33, 94.3%) than non-CL-related cases (13, 41.9%; *p* < 0.001; Table 3). Delayed corneal healing was noted in 29 (43.1%) cases, including 11 (31.4%) CL-related cases and 18 (58.1%) non-CL-related cases (*p* = 0.030; Table 3).

### Prognostic factors for visual outcomes and corneal healing

Univariable logistic regression identified several factors that were significantly associated with poor visual outcome (final CDVA of >0.6 logMAR), including age >50 years (OR = 10.52, *p* = 0.003), poor presenting CDVA <0.6 logMAR (OR = 8.87, *p* = 0.007), infiltrate size >3 mm (OR = 5.53, *p* = 0.007), and central ulcer location (OR = 7.32, *p* = 0.001; Table 3). Conversely, CL-related keratitis was associated with a lower risk of poor visual outcome (OR = 0.05, *p* < 0.001). In multivariable analysis, only CL-related keratitis remained independently associated with reduced odds of poor visual outcome (aOR = 0.13, 95% CI: 0.01–0.84; *p* = 0.046), with other factors such as age >50 years, poor presenting CDVA (>0.6 logMAR), infiltrate size >3 mm, and central ulcer location not showing any statistical significance (all *p* > 0.05; Table 4).

**Table 4:** Predictors for poor visual outcome (i.e., final CDVA ≥0.6 logMAR) in *Pseudomonas aeruginosa* keratitis, based on univariable and multivariable logistic regression analyses.

Predictors	Univariable analysis OR (95% CI)	*p*-value	Multivariable analysis^*^ aOR (95% CI)	*p*-value
Age >50 years	10.52 (2.62–71.24)	**0.003**	1.97 (0.24–18.17)	0.52
Female gender	1.27 (0.43–3.95)	0.66	–	–
Contact lens-related	0.05 (0.01–0.20)	**<0.001**	0.13 (0.01–0.84)	**0.046**
Presenting CDVA >0.6 logMAR	8.87 (2.21–59.98)	**0.007**	1.77 (0.25–15.05)	0.56
Hypopyon (present)	1.92 (0.62–6.46)	0.27	–	–
Infiltrate >3 mm	5.53 (1.71–21.78)	**0.007**	1.46 (0.26–7.92)	0.66
Central infiltrate	7.32 (2.32–26.63)	**0.001**	3.01 (0.67–14.92)	0.15

^*^Only significant factors in univariable analysis (*p* < 0.10) were included in the multivariable logistic regression model. Statistically significant values (*p* < 0.05) are in bold.

OR, odds ratio; CI, confidence interval; CDVA, corrected distance visual acuity.

In terms of corneal healing time, univariable analysis identified several poor prognostic factors for delayed corneal healing, including poor presenting CDVA >0.6 logMAR (OR = 4.51, *p* = 0.008), infiltrate >3 mm (OR = 4.85, *p* = 0.003), and central location (OR = 3.82, *p* = 0.011; Table 5). In contrast, CL-related infection (OR = 0.33, *p* = 0.032) was shown to be associated with a lower risk of poor corneal healing. Presence of hypopyon and systemic immunosuppression were not significantly associated with delayed corneal healing (both *p* > 0.05). After multivariable adjustment, none of the factors retained statistical significance (Table 5).

**Table 5:** Predictors for delayed corneal healing (i.e., >30 days to achieve complete corneal healing from presentation, or occurrence of uncontrolled infection or corneal perforation requiring corneal gluing, tectonic or therapeutic keratoplasty, and/or evisceration/enucleation) in *Pseudomonas aeruginosa* keratitis, based on univariable and multivariable logistic regression analyses.

Predictors	Univariable analysis OR (95% CI)	*p*-value	Multivariable analysis^*^ aOR (95% CI)	*p*-value
Age >50 years	2.35 (0.66–6.70)	0.10	1.12 (0.24–4.97)	0.88
Female	0.97 (0.36–2.62)	0.94	–	–
Contact lens-related	0.33 (0.12–0.89)	**0.032**	0.88 (0.19–4.13)	0.86
Presenting CDVA >0.6 logMAR	4.51 (1.56–14.60)	**0.008**	2.22 (0.57–8.92)	0.25
Hypopyon (present)	2.04 (0.73–5.91)	0.17	–	–
Infiltrate >3 mm	4.85 (1.74–14.62)	**0.003**	2.62 (0.71–9.89)	0.14
Central infiltrate	3.82 (1.39–11.15)	**0.011**	1.71 (0.47–6.18)	0.41
Systemic immunosuppression	2.46 (0.54–11.33)	0.25	–	–

^*^Only significant factors in univariable analysis (*p* < 0.10) were included in the multivariable logistic regression model. Statistically significant values (p < 0.05) are in bold.

OR, odds ratio; aOR, adjusted odds ratio; CI, confidence interval; CDVA, Corrected-distance-visual-acuity.

Systemic immunosuppression included patients with diabetes or those on systemic immunosuppressive drugs.

**Figure 1:**
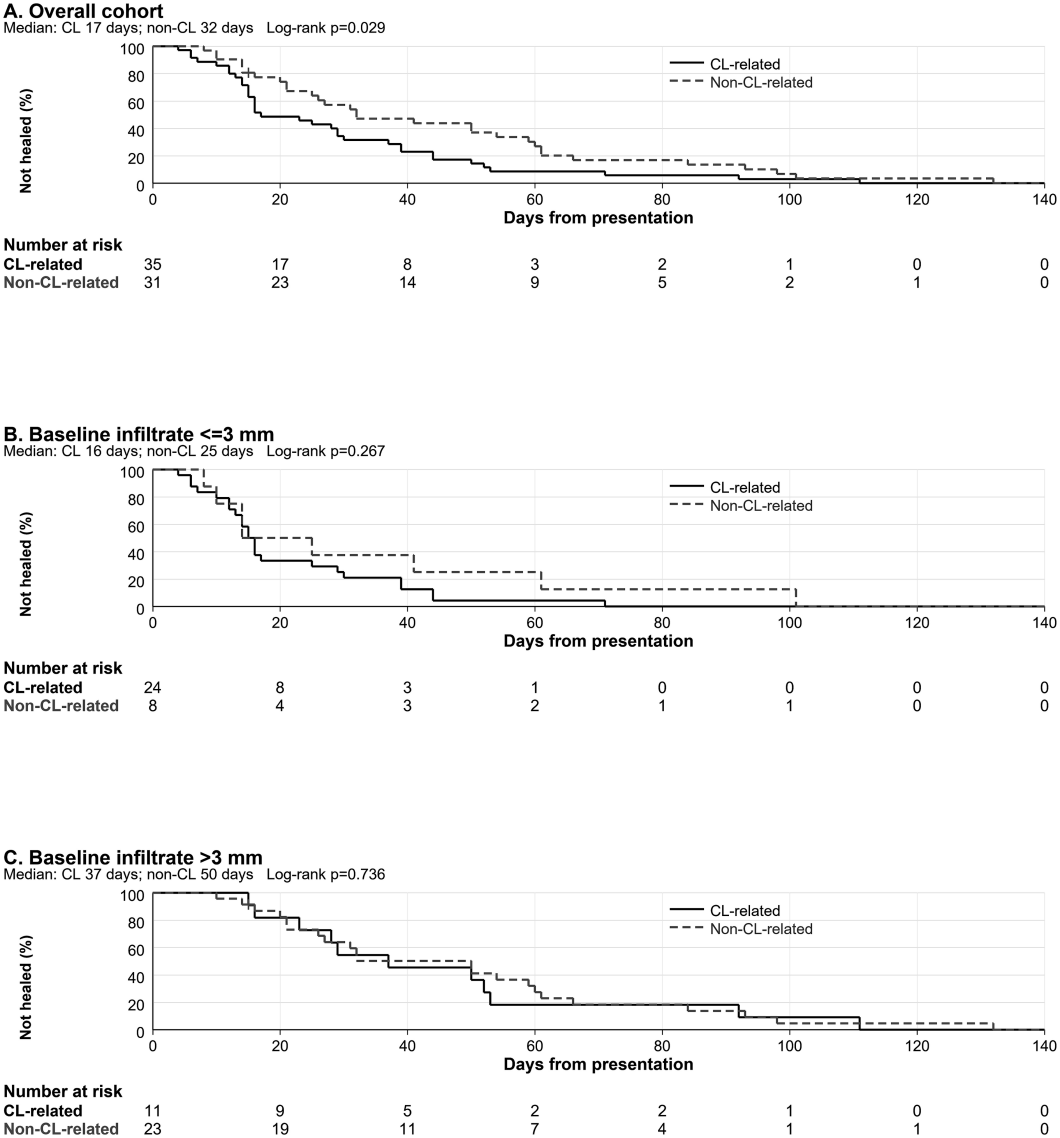
Kaplan–Meier curve showing time to complete corneal healing in contact lens (CL)-related and non-CL-related *Pseudomonas aeruginosa* keratitis. **(A)** Overall comparison between CL-related and non-CL-related cases. **(B)** Comparison restricted to cases with baseline infiltrate size ≤3 mm. **(C)** Comparison restricted to cases with baseline infiltrate size >3 mm. Differences between curves were assessed using the log-rank test.

Kaplan–Meier survival analysis demonstrated a significantly longer time to complete corneal healing in non-CL-related ulcers compared with CL-related cases (median 32 days vs. 17 days, log-rank *p* = 0.029) (Figure 1A). As the groups differed in baseline infiltrate size, additional analyses were stratified by infiltrate size. Amongst ulcers with an infiltrate size ≤3 mm, median healing time was 16 days in CL-related cases and 25 days in non-CL-related cases (log-rank *p* = 0.27; Figure 1B). Amongst ulcers with an infiltrate size >3 mm, median healing time was 37 days and 50 days, respectively (log-rank *p* = 0.74; Figure 1C). These findings suggest that the unadjusted difference in epithelial-healing time should be interpreted in the context of presenting disease severity.

## Discussion

IK remains a major cause of irreversible visual loss worldwide, and PA continues to attract particular concern due to its aggressive virulence, rapid epithelial destruction, and frequent association with hypopyon or corneal melt. CL wear is widely recognised risk factor for PA keratitis. Multiple studies have linked lens wear to microtrauma, intermittent hypoxia, contamination from water or lens cases, all of which facilitate PA penetration into the cornea (14, 20, 21). However, PA infection is not confined to CL wearers, and it often affects non–CL wearers who frequently have additional ocular or systemic diseases. In addition, the Asia Cornea Society Infectious Keratitis Study showed considerable geographical variations in the proportion of IK cases attributable to PA across participating centres, highlighting regional differences in microbial epidemiology (22).

In this cohort, around half of the cases (53%) occurred in CL users and the remaining cases occurred in non–CL wearers, emphasising that PA-related keratitis affects a wider general population. The overall clinical burden was considerable, with over 70% required admission for intensive therapy. When comparing the two groups, CL-related PA keratitis occurred in younger patients and presented with a less severe clinical phenotype. These cases had smaller epithelial defects and infiltrates, and better presenting vision. Symptom duration was numerically shorter in CL-related cases, although the difference did not reach statistical significance. By contrast, non-CL-related PA keratitis exhibited a distinct profile. These patients were significantly older (mean age 70.5 vs. 41.5 years), had a larger infiltrate, a higher proportion of central ulcer, more frequent hypopyon, and worse presenting CDVA. These observations are in keeping with reports from other regions showing that age, central ulcer location, and worse baseline vision are markers of more severe disease and poorer prognosis (4, 13, 21). Collectively, the clinical picture in this group resembles previously described high-risk microbial keratitis phenotypes.

Risk factors differed markedly between groups. Most CL-related cases lacked additional risk factors, whereas non-CL-related infections were frequently associated with OSD, systemic immunosuppression (including diabetes), previous corneal surgery, or topical corticosteroid use. These associations are consistent with previous studies (4, 11, 20, 23–25). Each of these factors has been shown to increase susceptibility to more severe microbial keratitis. OSD compromises epithelial integrity, promotes inflammation, and reduces corneal sensation, thereby delaying symptom recognition and causing stromal involvement (12, 26). Systemic immunosuppression weakens immune defences and has been linked to rapidly progressive keratitis (23). Prior corneal surgery reduces structural resilience and sensory feedback, making the eye more vulnerable to tissue breakdown during infection (27). Corticosteroid use can further reduce local immune responses and facilitate bacterial proliferation (12). Altered ocular surface microbiota in systemic disease, particularly diabetes, may further predispose to PA infection (28).

Delayed presentation may contribute to more severe infectious keratitis (3). Previous studies have shown that patients who present later often exhibit larger epithelial defects, stromal involvement, and higher rates of hypopyon, resulting into worse final vision (12, 26). Similar patterns have been reported from South Asia cohorts, where later referral was linked to poor visual outcome and prolonged healing (29). In our study, patients affected by non-CL-related PA keratitis presented later than those affected by CL-related PA keratitis, though the difference was not statistically significant.

Antimicrobial resistance has been increasing reported in PA keratitis in several regions, particularly in India, China and United States (2, 3). Reassuringly, antimicrobial susceptibility was high amongst isolates with available results. All isolates tested against amikacin were susceptible, while resistance to gentamicin and ciprofloxacin was identified in only one isolate each. Resistance proportions did not differ significantly between CL-related and non-CL-related cases. These findings suggest that differential susceptibility to these agents is unlikely to explain the observed differences in clinical outcomes.

Across the cohort, adults (>50 years), poor presenting CDVA (>0.6 logMAR), infiltrates >3 mm, and central ulcer location predicted worse vision on univariable analysis, consistent with prior United Kingdom and international studies (11, 30, 31). After adjustment, CL-related disease remained associated with lower odds of poor final CDVA. However, this observational association should not be interpreted as a causal protective effect of CL wear, particularly given the smaller ulcers and less severe baseline features in the CL-related group.

For corneal healing, poor presenting CDVA, larger infiltrate size, and central ulcer location were associated with delayed recovery, consistent with established predictors (30). However, none of these factors remained significant after multivariable adjustment. Kaplan–Meier analysis demonstrated slower corneal healing in non-CL-related cases (median 32 vs. 17 days), indicating a prolonged recovery in this group. However, when stratified by baseline infiltrate size, the difference in the rate of corneal healing between the two groups was not statistically significant within either infiltrate-size stratum. The unadjusted healing-time difference should therefore be interpreted in the context of baseline disease severity.

Our study presents the first United Kingdom-based comparative analysis of CL-related and non-CL-related PA keratitis. These findings highlight the need for greater clinical vigilance in patients with OSD, systemic immunosuppression and older patients presenting with non-CL-related PA keratitis. Early recognition, prompt microbiological investigation, and timely escalation of therapy may reduce the risk of corneal perforation, surgical intervention, and irreversible vision loss. Strengthening preventive strategies, particularly public awareness of CL hygiene and timely access to specialist care, may help reduce socioeconomic burden.

Several methodological limitations should be acknowledged. The retrospective design limited accuracy of behavioural data, including lens care, overnight wearing, and water exposure. The tertiary-care setting may introduce referral bias, and subtle inter-centre variability in admission thresholds, antimicrobial regimens, and follow-up intervals cannot be excluded.

Future research should include prospective multicentre studies with predefined standardised behavioural data collection and objective measures of epithelial healing (32). Integration of emerging technologies such as *in vivo* confocal microscopy, anterior-segment optical coherence tomography, molecular or AI-assisted microbial identification, inputted into unified electronic datasets for corneal and ocular surface disease, could enhance diagnostic precision and inter-centre comparability (33). Embedding these modalities within prospective study designs would strengthen causal inference and allow more granular delineation of organism-specific outcomes.

## Data Availability

The original contributions presented in the study are included in the article/Supplementary material, further inquiries can be directed to the corresponding author.
